# System- und Fertigkeitseinsatz in einem österreichischen Notarztsystem: retrospektive Studie

**DOI:** 10.1007/s00101-020-00820-8

**Published:** 2020-07-21

**Authors:** G. Prause, S. Orlob, D. Auinger, M. Eichinger, P. Zoidl, M. Rief, P. Zajic

**Affiliations:** Klinische Abteilung für Allgemeine Anästhesiologie, Notfall- und Intensivmedizin, Medizinische Universität Graz, Auenbruggerplatz 5, 8036 Graz, Österreich

**Keywords:** Notfallmaßnahmen, Effizienz, Inzidenz, Ausbildung, Kompetenz, Emergency measures, Efficiency, Incidence, Education, Competence

## Abstract

**Hintergrund:**

Die stetig wachsende Zahl der Notarztanforderungen und der geringe Anteil indizierter Einsätze führen zum Attraktivitätsverlust des Notarztdienstes, was sich vielerorts bereits durch nichtbesetzbare Notarztdienste bemerkbar macht. Vorliegende retrospektive Analyse evaluiert die Häufigkeit notärztlicher und medizinischer Maßnahmen in einem bodengebundenen Notarztsystem.

**Methode:**

Retrospektive Analyse anonymisierter Daten aus der Datenbank des Notarztstützpunkts LKH Univ.-Klinikum Graz. Die von Notärztinnen und Notärzten zwischen 2010 und 2018 absolvierten Einsätze wurden extrahiert, durchgeführte Maßnahmen evaluiert und je nach Schwierigkeitsgrad in 3 Kategorien aufgeschlüsselt: spezifische notärztliche Maßnahmen (Kategorie I), allgemein-medizinische Maßnahmen (Kategorie II), keine ärztliche Tätigkeit (Kategorie III). Die Häufigkeiten des Auftretens dieser Kategorien zwischen den Jahren wurden verglichen und Inzidenzen einzelner Maßnahmen pro 100.000 Einwohner errechnet.

**Ergebnisse:**

Im Beobachtungszeitraum wurden 15.409 Primäreinsätze und 322 Sekundärtransporte extrahiert und analysiert. Die jährliche Einsatzrate stieg beinahe kontinuierlich von 1442 Einsätzen 2010 auf 2301 Einsätze 2018. Bei 3687 (23,4 %) Stornierungen kam es zu 12.044 Patientenkontakten. Insgesamt wurden 2842 (18 %) Einsätze der Kategorie I, 7371 (47 %) Einsätze der Kategorie II sowie 5518 (35 %) Einsätze der Kategorie III verzeichnet. Die Häufigkeit für *notärztliche* Maßnahmen kann daher auf 157/100.000 Einwohner, die medizinischer Maßnahmen auf 409/100.000 Einwohner geschätzt werden.

**Schlussfolgerung:**

In einem Großteil aller Alarmierungen ist keine spezifisch *notärztliche* Maßnahme erforderlich. Somit erscheint das aktuelle Modell der präklinischen Versorgung nicht patientenorientiert und effizient. Der niedrige Anteil kritisch kranker bzw. schwer verletzter Patientinnen und Patienten führt bereits merkbar zum Attraktivitätsverlust bei den Notärzten und auch zu einer drohenden Qualitätsproblematik durch zu geringe Einsatzerfahrung und fehlendem Training.

## Abstract (en)

**Background:**

The continuous rise in calls for emergency physicians and the low proportion of indicated missions has led to a loss of job attractiveness, which in turn renders services in some areas unable to sufficiently staff units. This retrospective analysis evaluated the frequency of emergency and general medical interventions in a ground-based emergency physician response system.

**Methods:**

A retrospective analysis of anonymized data from the electronic documentation system of the emergency physician response unit at the Medical University of Graz was carried out. Calls answered by emergency physicians between 2010 and 2018 were extracted, measures carried out were evaluated and categorized into three groups: specific emergency interventions (category I), general medical interventions (category II) and no medical activity (category III). The frequency of occurrence of these categories was compared and incidences of individual measures per 100,000 inhabitants were calculated.

**Results:**

A total of 15,409 primary responses and 322 secondary transports were extracted and analyzed. The annual rate of system activation rose almost continuously from 1442 calls in 2010 to 2301 calls in 2018. The 3687 (23.4%) cancellations resulted in 12,044 patient contacts. Of these, 2842 (18%) calls were coded as category I, 7372 (47%) as category II and 5518 (35%) as category III. The frequency of specific emergency measures and general medical interventions was estimated at 157/100,000 and 409/100,000 inhabitants, respectively.

**Conclusions:**

No specific emergency physician interventions were required in the majority of call-outs. The current model of preclinical care does not appear to be patient-oriented and efficient. Furthermore, the low proportion of critically ill and injured patients already leads to a reduction in attractiveness for emergency physicians and may introduce the threat of quality issues due to insufficient routine experience and lack of training.

## Einleitung

Seit Jahrzehnten wird in Europa eine Diskussion über das optimale Notfallversorgungssystem geführt. Österreich hatte sich in den 1980er-Jahren zum „frankogermanischen“ Modell bekannt und stützt sich seitdem auf ein flächendeckendes bodengebundenes Notarztrettungssystem [10, 22]. Im Gegensatz dazu führen v. a. im angloamerikanischen Raum „paramedics“ (speziell ausgebildete qualifizierte Sanitäter) notfallmedizinische Erstversorgungen auf der Basis von Algorithmen und Leitlinien durch; präklinisch tätige Ärzte sind nur in Ballungszentren verfügbar und können nur für spezielle Indikationen angefordert werden.

**Figure 1:**
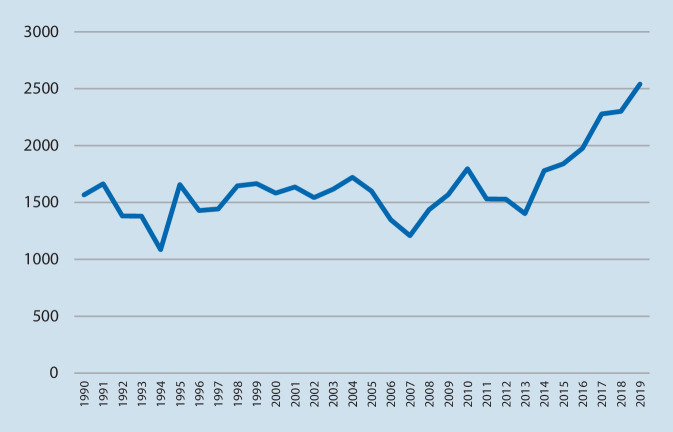

Die noch vor Jahren bestehenden gegensätzlichen Fronten scheinen sich langsam anzunähern. Etliche Studien belegen, dass die notärztliche Versorgung, v. a. im Bereich komplexer Notfallinterventionen, zu verbesserten patientenorientierten Ergebnissen führt [4, 7, 9]. Auf der anderen Seite müssen die Befürworter des frankogermanischen Modells des flächendeckenden Notarztwesens erkennen, dass das große „Angebot“ an Notarztmitteln zu einem kontinuierlichen, fast unkontrollierbaren Anstieg von Alarmierungen geführt hat. Mancherorts ist diese Zunahme so stark, dass sich die Einsatzfrequenzen im Vergleich zu jenen aus den 1990er-Jahren vervierfacht haben ([21]; Abb. 1). Dies ist weder mit dem Bevölkerungszuwachs [30] noch mit geänderten notfallmedizinischen Indikationen zu erklären [13, 21].

Eine Folge daraus ist die stetige Abnahme der Attraktivität der Notarzttätigkeit mit dem Effekt, dass notärztliche Dienste mancherorts schwerer bzw. teilweise gar nicht mehr besetzt werden können. Dies wird durch den manifesten Ärztemangel noch verstärkt. Dadurch erscheint es notwendig, die Sinnhaftigkeit und Effizienz der derzeitigen Verwendung notärztlicher Ressourcen im österreichischen Rettungsdienst kritisch zu hinterfragen, um die hochqualitative, medizinische Notfallversorgung nachhaltig gewährleisten und gleichzeitig die Verfügbarkeit notärztlicher Ressourcen bewahren zu können.

Diese Studie soll die Notarzteinsätze nicht als „indiziert oder nicht“ bewerten, sondern die tatsächlich von den Notärztinnen und Notärzten im Einsatz gesetzten Maßnahmen an einem bodengebundenen Notarztstützpunkt im Hinblick auf die Fragestellung untersuchen, ob für die Akutversorgung unbedingt die doch hochkomplexe Ausbildung zum Notarzt erforderlich war, um so die derzeitige Verwendung notärztlicher Ressourcen zu charakterisieren und zukünftige Verbesserungspotenziale aufzuzeigen.

## Patienten, Materialien und Methoden

Das Notarztsystem des LKH-Univ.-Klinikum Graz ist Teil des gesamten Versorgungskonzepts für den Großraum Graz und betreut einen Einzugsbereich von ca. 200.000 Menschen [23]. Bis zum Jahr 1993 versorgte ein Notarztsystem den gesamten Großraum Graz (>300.000 EW); im Jahr 2004 wurde für den Tagesbetrieb ein zweites Notarztsystem eingeführt und ab 2007 auch 24 h täglich ganzjährig betrieben.

Diese Studie ist eine retrospektive Analyse von Einsatzdaten des Notarztsystems des LKH Univ.-Klinikum Graz, welche anonymisiert aus der elektronischen Einsatzdatenbank extrahiert wurden. Der Notarztdienst wird von durchschnittlich 25 verschiedenen Notärztinnen und Notärzten interdisziplinär (Mitarbeiterinnen und Mitarbeiter der Univ.-Kliniken für Anästhesiologie und Intensivmedizin, Chirurgie, innere Medizin, Orthopädie und Traumatologie) besetzt.

Die Disposition des Notarztfahrzeugs erfolgt durch die zentrale Leitstelle der Rettungsorganisation. In dieser tätige Mitarbeiterinnen und Mitarbeiter haben zumindest eine Qualifikation als Rettungssanitäter gemäß österreichischem Sanitätergesetz [6] sowie eine Zusatzausbildung von etwa 250 h (Spezialkurs Leitstellendisposition), einschließlich praktischer Ausbildung an 20 Arbeitstagen. Im Beobachtungszeitraum erfolgte die Abfrage durch freies Interview.

### Ethische Implikationen

Die Ethikkommission der Medizinischen Universität Graz (IRB00002556) stimmte der Durchführung der Studie zu (Entscheidung 31-568 ex 18/19). Von der Notwendigkeit informierter Einverständnis („informed consent“) wurde abgesehen, da keine patientenbezogenen Studienmaßnahmen durchgeführt wurden.

### Datenquelle und -extraktion

Alle Daten wurden in der Routine des Einsatzes durch Notärztinnen und Notärzte mithilfe eines elektronischen Protokollsystems (MEDEA, Fa. iLogs, Klagenfurt, Österreich) erfasst. Für diese Studie wurden alle Einsätze vom 01.01.2010 bis 31.12.2018 mithilfe des dazugehörigen Exportwerkzeugs anonymisiert aus dem elektronischen Dokumentationssystem extrahiert.

Die Abfragen erfolgten einerseits nach gezielten Parametern bzw. andererseits aus der Kombination bzw. dem Ausschluss eingegebener Daten. So sind die Punkte Intubation, Beatmung, Reanimation, invasive Druckmessung und Ähnliches eigene Datenfelder, während sich der Begriff „Narkoseeinleitung“ aus der Kombination von Analgetika und/oder Sedativa und/oder Relaxans, kombiniert mit Beatmung, zusammensetzte. Der Parameter „nur venöser Zugang“ ergab sich aus dem Ausschluss jeglicher anderen Maßnahme, inklusive des Fehlens verabreichter Medikamente. Im Freitext wurden keine Maßnahmen dokumentiert.

Die Bearbeitung der Daten zum Zwecke der statistischen Analyse erfolgte mit kommerziell erhältlicher Tabellenkalkulationssoftware (Excel®, Microsoft Corp., Redmond, USA). Die Einsatzzahlen der letzten 30 Jahre wurden den Jahresstatistiken (Univ.-Klinik für Anästhesiologie und Intensivmedizin bzw. des Rettungsdienstes) entnommen.

### Datenbearbeitung und -analyse

Die extrahierten Einsätze wurden anhand einer vordefinierten Liste durchgeführter Maßnahmen klassifiziert (Tab. 1). Dabei wurden 3 Kompetenzkategorien definiert, um die Gesamtinvasivität der gesetzten Maßnahmen zu klassifizieren und den dafür erforderlichen Ausbildungsgrad zu bestimmen. Diese Kategorisierung erfolgte nach bestem Wissen um die erforderlichen Fertigkeiten der durchzuführenden Maßnahmen und auf der Basis der verfügbaren Evidenz für die akute Versorgung. Die Zuordnung zu diesen Kategorien erfolgte anhand der in Tab. 1 dargestellten Kriterien. Die Kategorien gliederten sich:

- *Kategorie 1 (notärztliche Maßnahmen)*: für gewöhnlich intensivmedizinische Maßnahmen, welche hoher notärztlicher Kompetenz bedürfen, um im präklinischen Bereich bei vorliegendem Beleg oder Hinweis auf Nützlichkeit bezüglich patientenorientierter Endpunkte zur Anwendung zu kommen;
- *Kategorie 2 (medizinische Maßnahmen)*: ärztliche, auch allgemeinmedizinische, Maßnahmen, sowie auch jene Fälle, in denen ein Transport in eine geeignete Krankenanstalt auch ohne (not)ärztliche Intervention möglich gewesen wäre;
- *Kategorie* *3 (keine medizinischen Maßnahmen)*: jene Fälle, in denen keine ärztliche Intervention erforderlich war.

**Table 1:** 

**Kategorie 1: notärztliche Maßnahmen**	
*Atemwegsmanagement*	Endotracheale Intubation
*Beatmung*	Invasive Beatmung
	Nichtinvasive Beatmung
*Gefäßzugänge*	Arterieller Zugang
*Interventionen*	Katecholamingabe
	Kardiopulmonale Reanimation
	Präklinische Narkoseeinleitung
	Behandlung des akuten Koronarsyndroms
	Transkutane Schrittmachertherapie
	Thorakozentese
**Kategorie 2: medizinische Maßnahmen**	
*Diagnostik*	12-Kanal-EKG (ohne Behandlung des akuten Koronarsyndroms)
*Gefäßzugänge*	Venöser Zugang (ohne Applikation von Medikamenten zur spezifischen Therapie)
*Interventionen*	Analgosedierung
	Leitsymptomorientierte Behandlung bei: Krampfanfall, Kollaps, Hypoglykämie, Atemwegsinfektionen, Kolikschmerzen, Bluthochdruck, Geburt, Brustschmerzen, Schlaganfall (ohne Maßnahmen der Kategorie 1)
**Kategorie 3: keine medizinischen Maßnahmen**	
*Interventionen*	Sanitätshilfliche Maßnahmen (Rettung, Sauerstoffgabe, Lagerung, Schienung)
	Todesfeststellung (keine Reanimationsmaßnahmen sinnvoll)
*Einsatzverlauf*	Stornierung bei Anfahrt
	Leerfahrten, Irreführungen

Allgemeine Einsatz- und Patientencharakteristika sowie Einsatzhäufigkeiten wurden mit üblichen Methoden der deskriptiven Statistik dargestellt. Die Anteile von Einsätzen der Kategorien 1, 2 und 3 wurden zwischen den ersten 6 (2010–2015) und letzten 3 (2016–2018) Jahren des Beobachtungszeitraums verglichen und mittels Chi-Quadrat-Test auf signifikante Unterschiede überprüft. Diese Analysen erfolgten in IBM® SPSS® Version 25 (Armonk, NY, USA). Mittelwerte sind als Median mit Interquartilsabstand (IQR) angegeben.

## Ergebnisse

**Figure 2:**
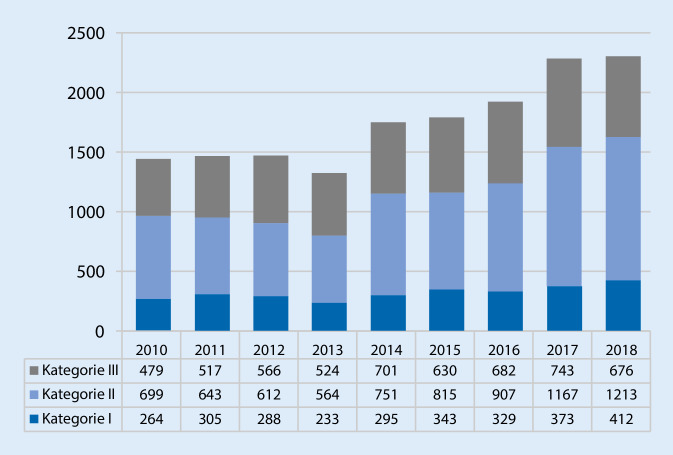

Im Beobachtungszeitraum vom 01.01.2010 bis zum 31.12.2018 wurden 15.731 Einsätze vom Notarztsystem LKH Univ.-Klinikum Graz absolviert; dies entspricht einer medianen Inanspruchnahme von 1722 Alarmierungen/Jahr. Eine beinahe fortwährende Steigerung der Einsatzzahlen war zu beobachten; während im Jahr 2010 noch 1442 Einsätze absolviert wurden, waren es 2018 bereits 2301 (Abb. 1). Den Verlauf der Einsatzzahlen der letzten 30 Jahre zeigt Abb. 2, wobei die Überführung in den 24-h-Betrieb (2003) kurzfristig ein „Einknicken“ der Verlaufskurve bewirkte.

Insgesamt wurden 15.409 Primäreinsätze und 322 (2,0 %) Sekundärtransporte absolviert. Das Notarzteinsatzfahrzeug wurde in 3687 (23,4 %) Fällen storniert; daraus resultierten 12.044 Patientenkontakte. Die betreuten Patientinnen und Patienten waren im Median 66 (41 bis 81) Jahre alt und mehrheitlich männlich (*n* = 6303, 53,3 %).

Von allen Alarmierungen wurden 2842 (18 %) Einsätze der Kategorie I, 7371 (47 %) der Kategorie II sowie 5518 der Kategorie III (35 %) verzeichnet. Würde man das Anlegen einer Venenverweilkanüle und Applikation einer Infusion auch als nichtärztliche Maßnahme einstufen, würde sich der Anteil derartiger Einsätze (Kategorie III – kein Arzt erforderlich) schon auf etwa 50 % erhöhen.

Der Anteil von Einsätzen der Kategorie I veränderte sich über die Zeit hinweg eher rückläufig; während er in den ersten 6 Jahren des Beobachtungszeitraums noch bei 18,7 % lag, war er in den letzten 3 Jahren 17,1 % (*p* = 0,011). In der Kategorie II war eine signifikante Zunahme von Einsätzen über die Zeit zu verzeichnen; in den ersten zwei Dritteln der Beobachtungszeit lag der Anteil bei 44,3 %, im letzten Drittel schon bei 50,6 % (*p* < 0,001). Auch der Anteil an Einsätzen der Kategorie III änderte sich signifikant über den Zeitraum, der höchste Anteil war jedoch in der Mitte des Beobachtungszeitraums festzustellen; im Vergleich der ersten 6 mit den letzten 3 Jahren fiel der Anteil von 37,0 auf 32,3 % (*p* < 0,001) (Abb. 1).

Die genaue Analyse der Tätigkeiten, geordnet nach den Kategorien, die Inzidenz der gesetzten Maßnahmen auf Basis von 100.000 Einwohnern/Jahr sowie die Berechnung der einzelnen Tätigkeiten bezogen auf einen einzelnen Notarzt sind in Tab. 2 aufgelistet. Ein erheblicher Teil der Patientinnen und Patienten – v. a. der Kategorie 1 – erhielt mehr als eine Maßnahme, sodass die Summe der Tätigkeiten höher als die Patientenzahl ist.

**Table 2:** 

	Anwendungen	Pro Jahr	Inzidenz 100.000 EW	Inzidenz/ Notarzt
**Kategorie 1: notärztliche Maßnahmen**	*2842*	*315,8 Pt*	*157,8*	*12,6*
Endotracheale Intubation	1226	136,2	68,1	5,4
Invasive Beatmung	1509	167,7	83,8	6,7
Nichtinvasive Beatmung	506	56,2	28,1	2,2
Arterieller Zugang	710	78,9	39,4	3,2
Katecholamingabe	788	87,6	43,8	3,5
Kardiopulmonale Reanimation	1028	114,2	57,1	4,6
Präklinische Narkoseeinleitung	646	71,8	35,9	2,9
Behandlung des akuten Koronarsyndroms	621	69,0	34,5	2,8
Transkutane Schrittmachertherapie	22	2,4	1,2	0,1
Thorakozentese	38	4,2	2,1	0,2
**Kategorie 2: medizinische Maßnahmen**	*7371*	*819,0*	*409,5*	*32,8*
12-Kanal-EKG	3867	429,7	214,8	17,2
Venöser Zugang, Infusion	2412	268	134	10,7
Analgosedierung	2817	313,0	156,5	12,5
Leitsymptomorientierte Behandlung	4866	540,7	270,3	21,6
*Kategorie 3: keine medizinischen Maßnahmen*	*5518*	*613,1*	*306,6*	*24,5*
Sanitätshilfliche Maßnahmen (Rettung, Sauerstoffgabe, Lagerung, Schienung)	1378	153,1	76,6	6,1
Todesfeststellung	453	50,3	25,2	2,0
Stornierung, Leerfahrt, Irreführung	3687	409,7	204,8	16,4
*Gesamtsumme*	*15.731*	*1747,9*	*873,9*	*69,9*

Dargestellt als Gesamtzahl, gebrochen auf ein Jahr

Die Inzidenz der Maßnahmen wurde pro Jahr und 100.000 EW berechnet, sowie pro Notarzt und Jahr

## Diskussion

Die Anzahl der Notarztalarmierungen hat sich seit den Anfängen in den 1980er-Jahren mehr als vervierfacht [21, 22], während sich im Gegensatz dazu die zugrunde liegende Einwohnerkennzahl im gleichen Zeitraum nur um etwa 18 % erhöht hat [30]. Die aktuellen Zahlen stammen vom lokalen Notarztsystem, welches sogar eine gewisse Sonderstellung einnimmt, da es österreichweit sowohl das größte Einzugsgebiet (ein Notarzt für 200.000 Menschen) als auch die geringste Inzidenz an Notarztalarmierungen aufweist [23]. Umgerechnet auf die österreichische Gesamtbevölkerung muss man auf Basis der in der vorliegenden Arbeit identifizierten Rate spezifisch notärztlicher Interventionen (157 auf 100.000) annehmen, dass pro Jahr etwa 13.000 Patientinnen und Patienten einer notärztlichen Versorgung bedürfen, deren Sinnhaftigkeit mit hoher Evidenz belegt ist (Kategorie I). Tatsächlich wurden im Jahr 2014 jedoch schon über 200.000 Einsätze absolviert; Tendenz nach wie vor steigend. Daraus lässt sich ableiten, dass derzeit deutliche Optimierungspotenziale in der Verwendung notärztlicher Ressourcen im österreichischen Rettungsdienst bestehen.

Die angewandte Kategorisierung ist eine retrospektive, kritische Analyse der von den Notärzten durchgeführten Maßnahmen und basiert auf den Kriterien der Effektivität und der dazu erforderlichen Kompetenz. Die optimale präklinische Versorgung z. B. mit Narkoseeinleitung, Intubation und Beatmung erfordert eine hochqualifizierte Ausbildung und auch eine entsprechende Erfahrung, die am ehesten mit intensivmedizinischen Standards vergleichbar ist. Diese Kategorie erfordert die spezifische notärztliche Kompetenz; die Maßnahmen sind auch für den präklinischen Bereich gut durch Studien abgesichert [4, 8, 9, 18] und können nicht von anderwärtigen medizinischen Einrichtungen übernommen werden.

Aus der niedrigen Anwendungshäufigkeit ergibt sich auch die Gefahr mangelnder Routine im Notarztdienst. Verglichen mit gängigen Anforderungen der Fachgesellschaften für eine adäquate Anwendung der Intubation [19, 27, 29], Beatmung oder Narkoseeinleitung zeigt sich, dass die alleinige Tätigkeit im prähospitalen Notarztdienst für eine derartige Qualifikation nicht ausreichend sein kann [14, 25, 28]. Gerade aber für diese hochspezifischen Tätigkeiten ist die adäquate Routine in der Anwendung entscheidend, um zu verbesserten patientenbezogenen Ergebnissen zu führen [7, 18]. Die aktuelle Novelle des österreichischen Ärztegesetzes trägt diesem Gedanken bereits Rechnung und sieht die notärztliche Ausbildung nur an Lehrkrankenanstalten vor, an welche Notarztsysteme angeschlossen sind (BGBl GPXXVI RV 385 AB 438, S 57; BR: 10081 AB 10116, S. 888).

Demgegenüber ergeben sich aus den Zahlen eindeutige Hinweise, dass offensichtlich ein großer Bedarf an präklinischer medizinischer Versorgung besteht, die zwar zeitkritisch als Notfall angefordert wird, wo aber keine spezifisch notärztlichen Maßnahmen erforderlich sind (Kategorie II). Der Notarzt ist oftmals der Ersatz für fehlende Infrastrukturen (Hausärztemangel) bzw. Ausbildungsdefizite im Rettungsdienst.

In Österreich besteht eine europaweit außergewöhnliche Situation, in welcher der Rettungsdienst zum überwiegenden Teil von Ehrenamtlichen und Zivildienstleistenden bewerkstelligt wird, die mehrheitlich eine Ausbildungszeit von 260 h (Rettungssanitäterinnen und Rettungssanitäter) ohne klinischen Teil einer Ausbildung aufweisen [24]. Auch die weiterführenden Ausbildungsprogramme für Notfallsanitäterinnen und Notfallsanitäter mit insgesamt 800 h (Notfallkompetenzen) sind nicht mit dem in vielen Ländern bestehenden Berufsbild von Sanitäterinnen und Sanitätern vergleichbar [6, 26]. In Österreich gibt es nur wenige Rettungsdienstbereiche (beispielsweise Wien, Graz und Innsbruck), in denen Notfallsanitäterinnen und Notfallsanitäter mit Notfallkompetenzen zu speziell definierten Einsätzen entsandt werden. Dass derartige Versorgungsmodelle in der Lage sind, die Alarmierungshäufigkeit der regionalen Notarztsysteme deutlich zu reduzieren, konnte in einer Studie nachgewiesen werden [23]. Das Notarztsystem Graz als 3‑stufiges Versorgungsmodell (Sanitäter – Notfallsanitäter mit Notfallkompetenz – Notarzt) weist darin 851 Einsätze/100.000 Einwohner auf, im Gegensatz zum Stützpunkt Wiener Neustadt mit 2‑stufiger Versorgung (Sanitäter – Notarzt), wo mehr als 3000 Einsätze/100.000 Einwohner absolviert wurden.

Die Kategorisierung erfolgte aus dem Blickwinkel, welche Maßnahmen welche ärztliche Qualifikation erfordern, und sind nicht zwingend eine Bewertung eines Einsatzes. So betrachten die Autoren beispielsweise den Fieberkrampf als absolut „notarztpflichtigen“ Einsatz; die gesetzte Maßnahme wurde vielfach bereits von der Mutter oder vom Hausarzt durchgeführt und erfordert somit in der Auswertung keine zwingend notärztliche Kompetenz (Kat. 2). Ebenfalls gibt es regionale Unterschiede, inwieweit z. B. die Feststellung des Todes (Unterlassung der Reanimationsmaßnahmen) eine ärztliche Aufgabe darstellt; im lokalen System führt der Notarzt keine Totenbeschau durch, und auch die Zwangsunterbringung ist nicht Aufgabe des lokalen Notarztsystems, sondern wird durch Amtsärzte bewerkstelligt. Demnach lässt sich auch die geringere Inzidenz an Reanimationen bzw. Todesfeststellungen im Vergleich zum German Resuscitation Registry (GRR) [11] durch das Modell der „abgestuften Notfallversorgung“ und der Einbeziehung des ärztlichen Bereitschaftsdienstes erklären.

Die Ursachen der zunehmenden Einsatzzahlen sind sicher vielfältig und mit der retrospektiven Analyse allein nicht nachvollziehbar. Es ist aus der Notarztdatenbank nicht auszuwerten, wer die Alarmierung ausgelöst hat, ob schon andere medizinische Kräfte (Hausarzt) angefragt wurden, bzw. ob die zuvor anwesende Rettungsmannschaft den Notarzt nachgefordert hat.

Der Trend zu höheren Notarzteinsatzzahlen ist in vielen europäischen Ländern zu beobachten; teilweise wurden Steigerungen bis zu 300 % berichtet [1, 12]. Gries et al. haben im Jahr 2013 in Deutschland eine Einsatzhäufigkeit von 2590 Einsätzen/100.000 Einwohner und Jahr aufgezeigt; eine Untersuchung zum Notarztdienst in Bayern des Instituts für Notfallmedizin und Medizin-Management aus dem Jahr 2010 hat 2820 Einsätze/100.000 Einwohner ausgewiesen [15]; Bader et al. haben für den Rettungsdienstbereich Leipzig eine Einsatzhäufigkeit von 5800 Einsätzen/100.000 Einwohner berichtet [1]. Auch in der Schweiz wurde innerhalb von 10 Jahren ein Anstieg der Rettungseinsätze um 40 % bemerkt [20]. Lediglich Bollinger et al. haben im Jahr 2015 eine im Vergleich zu den österreichischen Einsatzzahlen niedrigere Häufigkeit von 1940 Einsätzen/100.000 Einwohner gezeigt [2] Überproportionale Anstiege von Notfalleinsatzzahlen und sinkende Interventionsraten verzeichnen aber auch jene Länder, deren Rettungssysteme primär auf nichtärztlichen Mitarbeiterinnen und Mitarbeitern („Paramedics“) beruhen [17, 31]. Evans berichtete 2012 auch in England von dem Phänomen, dass die Notfallalarmierungen stetig zunehmen, aber in nur knapp 10 % aller Notrufe tatsächlich lebensbedrohliche Situationen vorlagen [9].

Die Autoren sehen in der Entwicklung die Gefahr, dass der Notarzt als „Arzt für alle Fälle“ zunehmend Aufgaben zugeteilt bekommt, die nicht seinem eigentlichen Portfolio („Versorgung lebensbedrohlich erkrankter/verletzter PatientInnen“) entsprechen. Diese auch international festzustellenden Probleme [3, 15, 21] haben die Bereitschaft v. a. erfahrener Ärztinnen und Ärzte sinken lassen, am Notarztdienst teilzunehmen. Wie aus diversen Pressemitteilungen zu entnehmen, gibt es in Österreich vielerorts Probleme in der Besetzung von Notarztdiensten. Ein denkbarer Lösungsansatz zur Minimierung von Notarztanforderungen – zumindest der physikalischen Ausfahrten – wäre der bereits in manchen Regionen Deutschlands implementierte „Telenotarzt“ [5]. Der Vorteil würde in diesen Fällen sicher in der schnelleren Verfügbarkeit des Notarztmittels liegen – derartige Projekte sind derzeit in Österreich nicht vorhanden.

### Limitationen

Diese Studie beruht auf der retrospektiven Analyse prospektiv im Rahmen der klinischen Routine erhobener und dokumentierter Daten und unterliegt daher allen Limitationen retrospektiver Datenanalysen. Die Datengewinnung ist direkt abhängig von der Dokumentationsgenauigkeit, weshalb v. a. die Datenqualität für Parameter, die nach Ausschlusskriterien erfasst wurden, reduziert sein kann. Die Generalisierbarkeit auf Landes- und Bundesebene sowie darüber hinaus ist durch die Auswertung von Daten aus ausschließlich einem Notarztsystem mit einem urbanen Einzugsgebiet möglicherweise limitiert. Die Hochrechnung auf die österreichische Bevölkerung erfolgt auf der Basis einer Erhebung aus dem Jahr 2014, während die Analyse der Maßnahmen über 9 Jahre erfolgt ist. Außerdem hat diese Studie nur die Einsätze mit Alarmierung des Notarzteinsatzfahrzeugs betrachtet und nicht das Rettungsdienstsystem im Gesamten. Das in Graz praktizierte Modell der „abgestuften Notfallversorgung“ [23] mag eine weitere Limitation hinsichtlich der Generalisierbarkeit der Ergebnisse darstellen.

### Zusammenfassung

Die Analyse der von den Notärztinnen und Notärzten durchgeführten Maßnahmen zeigte, dass spezifische notärztliche Maßnahmen nur mit einer geringen Inzidenz erforderlich sind. In etwa 30 % der Einsätze sind medizinische Standardmaßnahmen ausreichend; in der Hälfte der Fälle ist keine ärztliche Therapie notwendig.

### Schlussfolgerung

Österreich hat eine sehr hohe Dichte an Notarztsystemen, mit einer kontinuierlich steigenden Zahl an Einsätzen. Diese als hoch-positiv verkaufte Gegebenheit hat neben den hohen Kosten auch Schattenseiten. So ist zwar die Flächenabdeckung und Verfügbarkeit sehr hoch, der Anteil indizierter und ärztlich fordernder Einsätze jedoch ständig im Sinken begriffen. Wenn man aus den Zahlen des Notarztsystems Graz auf die österreichische Gesamtbevölkerung hochrechnet, erhält man als Ergebnis, dass der Anteil jener Notarzteinsätze, bei denen eine notärztliche Kompetenz mit hoher belegbarer Evidenz das Outcome des Patienten verbessert, österreichweit vermutlich in einem Bereich von rund 7 % liegt. Eine bereits spürbare Folge ist die mangelnde Bereitschaft hochqualifizierter Kollegen für diese Tätigkeit. Des Weiteren besteht die Gefahr, dass die verfügbaren Notärzte nicht über jene Erfahrung verfügen, die für die Versorgung schwer verletzter oder erkrankter Notfallpatientinnen und -patienten erforderlich ist. Allerdings zeigen diese Zahlen auch deutlich auf, dass die in manchen Ländern bestehenden Versorgungskonzepte mit „Berufsnotärzten“ in Österreich nicht sinnvoll erscheinen, da aufgrund der geringen Inzidenz die im Notfall erforderlichen Fertigkeiten im Rettungsdienst weder erlernt noch trainiert werden können.
